# Multilevel Safety Climate and Safety Performance in the Construction Industry: Development and Validation of a Top-Down Mechanism

**DOI:** 10.3390/ijerph13111100

**Published:** 2016-11-08

**Authors:** Ran Gao, Albert P.C. Chan, Wahyudi P. Utama, Hafiz Zahoor

**Affiliations:** Department of Building and Real Estate, The Hong Kong Polytechnic University, Hung Hom, Kowloon, Hong Kong 999077, China; albert.chan@polyu.edu.hk (A.P.C.C.); wahyudi.utama@connect.polyu.hk (W.P.U.); zahoor.khan@connect.polyu.hk (H.Z.)

**Keywords:** construction safety, safety climate, safety performance, structural equation modeling

## Abstract

The character of construction projects exposes front-line workers to dangers and accidents. Safety climate has been confirmed to be a predictor of safety performance in the construction industry. This study aims to explore the underlying mechanisms of the relationship between multilevel safety climate and safety performance. An integrated model was developed to study how particular safety climate factors of one level affect those of other levels, and then affect safety performance from the top down. A questionnaire survey was administered on six construction sites in Vietnam. A total of 1030 valid questionnaires were collected from this survey. Approximately half of the data were used to conduct exploratory factor analysis (EFA) and the remaining data were submitted to structural equation modeling (SEM). Top management commitment (TMC) and supervisors’ expectation (SE) were identified as factors to represent organizational safety climate (OSC) and supervisor safety climate (SSC), respectively, and coworkers’ caring and communication (CCC) and coworkers’ role models (CRM) were identified as factors to denote coworker safety climate (CSC). SEM results show that OSC factor is positively related to SSC factor and CSC factors significantly. SSC factor could partially mediate the relationship between OSC factor and CSC factors, as well as the relationship between OSC factor and safety performance. CSC factors partially mediate the relationship between OSC factor and safety performance, and the relationship between SSC factor and safety performance. The findings imply that a positive safety culture should be established both at the organizational level and the group level. Efforts from all top management, supervisors, and coworkers should be provided to improve safety performance in the construction industry.

## 1. Introduction

The construction industry is recognized as one of the most dangerous industries in the world [1]. The complexity of the construction procedures and the temporary character of the projects exposes front-line workers to dangers and accidents, and makes safety a rather complex issue [2]. At a rough estimate, the construction industry accounts for 30%–40% of fatal injuries, although it employs only 7% of the world’s workforce [3]. A large aggregation of studies have focused on construction safety and contributed to the improvement of safety performance [4]. A literature review summarized four key topics about construction safety over the last three decades, which are causes of construction accidents, the influence of management on accidents and accident prevention, safety in design, and safety climate and safety culture [2].

Safety climate is derived from organizational climate, and describes workers’ perceptions of the value of safety in their work environment [5]. Originally defined by Zohar in 1980, safety climate is described as “a unified set of cognitions regarding the safety aspects of the organization”, which “reflects employees’ shared perceptions about the relative importance of safe conduct in their occupational behavior”. Safety climate has been confirmed to be a predictor of safety performance in substantial studies [6,7,8]. Desai et al. [9] identified a positive relationship between safety climate and minor accidents, and no significant relationship was discovered between safety climate and major accidents. Clarke [6] conducted a meta-analysis with 32 published papers and identified a positive relationship between safety climate and safety behaviors. In the construction industry, insufficient safety precautions and weak safety climate are also deemed to be major causes of the high industrial accident rates and unsafe behaviors [7,10]. The complicated characteristics of construction provide rather complex conditions for the consideration of safety climate and pose challenges to safety climate research within this particular industry [11].

Melia et al. [12] split up safety climate in the construction sector into multilevel variables (i.e., the organizational safety response, the supervisors’ safety response, the coworkers’ safety response, and the workers’ safety response), and examined a psychosocial sequence of relationships among these safety responses with regression analysis. Compared to regression analysis, the structural equation modeling (SEM) method could analyze data with consideration of their structural complexity and permission of study on relationships among each factors concurrently [13]. Based on a similar framework to the study of Melia et al. [12], Brondino et al. [13] used the SEM method to test a model on the relationships between organizational and group safety climate and safety performance in the manufacturing industry.

**Figure 1:**
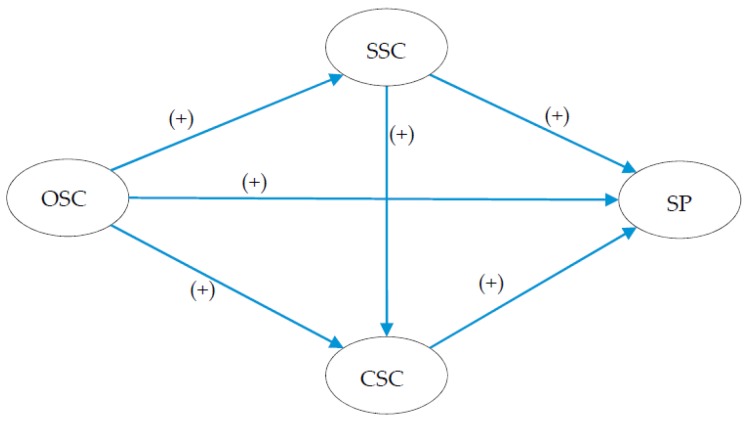
Research model and hypotheses. OSC: organizational safety climate, SSC: supervisor safety climate, CSC: coworker safety climate, SP: safety performance.

Similar to Brondino et al. [13], the current study seeks to explore the underlying mechanisms of the relationship between multilevel safety climate and safety performance using the SEM method, but in the construction industry. Distinct from Brondino et al. [13], different safety climate factors even from the same level are considered to be different constructs in the current study. To be specific, this paper aims to study how particular safety climate factors of one level affect those of other levels, and then affect safety performance from the top down. A model unveiling underlying mechanisms of the relationship between multilevel safety climate and safety performance would be useful for safety professionals to evaluate, supervise, and improve safety performance in construction projects. This model supposes sequent effects of safety climate factors of the organizational level on safety performance through safety climate factors at the supervisors’ and coworkers’ level. The following hypotheses were proposed according to the research of Melia et al. [12] and Brondino et al. [13], and the proposed relationships among factors are shown in Figure 1.

H1: Organizational safety climate (OSC) factors are positively related to supervisor safety climate (SSC) factors and coworker safety climate (CSC) factors.

H2: SSC factors mediate the relationship between OSC factors and CSC factors.

H3: SSC factors mediate the relationship between OSC factors and safety performance (SP).

H4: CSC factors mediate the relationship between OSC factors and SP.

H5: CSC factors mediate the relationship between SSC factors and SP.

## 2. Literature Review

### 2.1. Safety Climate in Construction

Among studies on safety climate, many scholars have conducted factor analyses to identify its distinct structures and dimensions [1,11,14,15]. In the construction industry, the number of safety climate dimensions varies from two [15] to fifteen [14]. Management’s commitment to safety, workers’ involvement in safety, and safety rules and procedures are the three most frequent safety climate factors in the construction industry [16]. Many studies have considered safety climate as a multilevel concept [12,13,17,18,19]. Zohar himself extended the original definition of safety climate in a longitudinal manner, by adding a group-level safety climate to the previous organizational safety climate [18]. The basic proposition of this development was that regulations were formulated at the organization level and implemented at the group level, and thus, safety climate could be formed from top management’s policy actions as well as front-line supervisors’ practical actions. In the construction industry, employees work in small groups and report to an appointed supervisor. Communication with supervisors represents to workers the real priority of safety through the practices supervisors implement regarding company safety regulations and the resolution of conflicts between safety and productivity [11], and the supervisor’s safety climate is thus formed. Lingard et al. [20] confirmed the existence of supervisor’s safety climate within the road construction and maintenance organization, and discovered that supervisory personnel, such as foremen and gangers, played a major role in affecting safety performance in workgroups. Besides supervisor’s safety climate, another potential dimension of group-level safety climate is coworkers’ safety climate, which is the extent to which workers care about their coworkers’ safety [12,13,21]. Coworkers give information, provide care, and act as role models in the work environment. Their behaviors influence workers’ task performance beyond supervisors’ behaviors [13]. Compared to managers and supervisors, coworkers are closer and larger in number. Workers tend to develop clear safety beliefs through exchanges with coworkers.

### 2.2. Safety Performance

Safety performance mainly includes two categories: safety outcomes and safety behaviors [22]. Safety outcomes provide historical information on bottom-line indicators of safety performance, which is traditionally measured by statistical data such as accidents and injuries [23,24]. Safety performance is also described as the actual safety behaviors that individuals performed at work, and is classified into safety compliance and safety participation [5,25]. Safety compliance describes safety-related behaviors required by the organization to be carried out by individuals to keep the workplace safe. Safety participation depicts voluntary safety-related behaviors that may not directly work on personal safety but help to develop an organizational context to support safety [5,26,27].

### 2.3. Relationship between Safety Climate and Safety Performance

The relationship between safety climate and safety performance has been learnt in substantial studies [12,13,26,28,29,30]. Morrow et al. [31] explored the relationship between different facets of safety climate and safety behaviors. Three facets including work-safety tension, management, and coworkers were considered, and the former one was found to be most strongly related to unsafe behaviors when compared with the latter two. Siu et al. [28] investigated the relations among safety climate, psychological strains, and accident rates, and found that psychological strains is a mediator of the relationship between safety climate and accident rates. Huang et al. [29] discovered that employee safety control was a mediator of the relationship between safety climate and self-reported injury. Griffin and Neal [26] identified that safety knowledge and safety motivation mediated the link between safety climate and safety performance (i.e., safety compliance and safety participation). Guo et al. [30] extended Griffin and Neal’s research and tested an integrative model in the construction industry to understand the mechanisms that explain how particular safety climate factors affect workers’ safety performance through individual factors (i.e., safety knowledge and safety motivation).

## 3. Research Methods

### 3.1. Questionnaire Design

To test the proposed hypotheses, a questionnaire survey approach was applied for data collection. The questionnaire was composed of three parts. The first part consisted of 19 questions asking the personal particulars of the participants. The second part was a 38-item multi-level safety climate scale based on a similar structure to Melia et al. [12] and Brondino et al. [13]. To conduct data analysis of different levels separately, as well as to carry out an integrated study of the relationships among them, the workers’ perceptions of safety climate from various levels including top management level, supervisor level and coworker level were measured in this study. The safety climate related to top management was evaluated with the organizational safety climate scale developed by Zohar and Luria [19] with 16 items which focus on the attitudes and activities of top management regarding safety management. For example, a sample item was “Top management is strict about working safely when work falls behind schedule”. The safety climate related to supervisor was measured with a 10-item scale derived from Zohar [18]. For example, a sample item was “My supervisor says a good word whenever he sees a job done according to the safety rules”. The safety climate related to coworkers was measured with a 12-item safety climate scale revised from Brondino et al. [13]. For example, a sample item in this scale was “My coworkers emphasize safety procedures when we are working under pressure”. The third part of the questionnaire was the safety performance scale. Safety performance in this study was considered to be the actual safety behaviors that individuals perform at work. A six-item safety performance scale developed from Neal and Griffin [25] was employed to measure these actual safety behaviors. Within the six items, three items were related to safety compliance, which were “I use all the necessary safety equipment to do my job”, “I use the correct safety procedures for carrying out my job”, and “I ensure the highest levels of safety when I carry out my job”. The other three items were related to safety participation, which were “I promote the safety program within the organization”, “I put in extra effort to improve the safety of the workplace”, and “I voluntarily carry out tasks or activities that help to improve workplace safety”. A five-point Likert scale was adopted to measure the response to each item from 1 to 5, in terms of strongly disagree, disagree, neutral, agree, and strongly agree, respectively.

The questionnaire was initially designed in English. Chinese versions and Vietnamese versions were obtained through translation. To guarantee semantic reliability, non-English versions had subsequently been translated back into English by a different translator team. Ambiguous translations were discussed and revised by the two translator teams. A panel of experts, comprising seven scholars and nine practitioners, were invited to make some suggestions to assure that the research content were exactly expressed and in line with practice situations. Several minor revisions were consequently made to the expression of the questions and the structure of the questionnaire.

### 3.2. Participants and Procedure

The data were collected from six construction sites in Vietnam in May 2015. All these projects were contracted by Chinese international contractors. To guarantee accurate responses from front-line workers and to encourage widespread participation from the investigated projects, the researchers went to the aforesaid construction sites and coordinated with the workers face-to-face with the help of interpreters. The research aims and objectives were conveyed to the participants clearly. The workers were assured that their participation was voluntary, all replies were anonymous and confidential, and no information would be disclosed to their supervisors or coworkers.

### 3.3. Data Analysis

In the current study, the exploratory factor analysis (EFA) was used to identify the potential multilevel safety climate factors. These factors were further verified and relationships among these factors were investigated by the SEM. Data were randomly split in two parts in the SPSS 17.0 (IBM, New York, NY, USA) for Windows software package. Approximately half of the data were used to conduct EFA in the SPSS 17.0 for Windows software package and the remaining data were submitted to SEM in the Analysis of Moment Structures (AMOS) version 17.0 (IBM, New York, NY, USA).

#### 3.3.1. Exploratory Factor Analysis

EFA and confirmatory factor analysis (CFA) are two discrete kinds of factor analysis. The originally defined factor analysis has now come to be called EFA. It is a powerful method to reduce variable complexity to greater simplicity by summarizing a larger quantity of variables to a smaller quantity of factors [32]. EFA allows the analysis to be concentrated on the principal components in order to acquire knowledge about dynamics of their relationships. In the current study, EFA was used to identify the factor structure of safety climate firstly. With understanding of the factor structure, SEM was then conducted in order to investigate the relationship among different safety climate factors and safety performance. Before EFA, both the Kaiser-Mayer-Olkin (KMO) measure of sampling accuracy and Barlett’s test of sphericity were conducted to evaluate the appropriateness of using the EFA method in this study. As a frequently-used extraction method whenever EFA are conducted, principal component analysis (PCA) was selected for data extraction in the current research. In this method, variables are put together according to their mutual correlations and then combined to a certain number of components [33]. To find out the number of factors that should be extracted and interpreted, parallel analysis was conducted in addition to Kaiser’s criterion and scree test. According to Pallant [34], parallel analysis is more accurate for determining the number of factors to be interpreted as the other two methods have a tendency to overrate the number of factors. The Oblimin oblique rotation method was used to interpret latent variables underlying a factor due to the potential correlations among these factors. The threshold of 0.50 was considered to be the minimum factor loading when determining an item to load on a latent factor [35].

#### 3.3.2. Structural Equation Modelling

SEM was conducted to test the potential theoretical relationships among different safety climate factors and safety performance in the current study. SEM usually contains a measurement model that defines latent variables with several observed variables, and a structural model that studies the relationships between latent variables. The SEM method was chosen because it could estimate the theoretical relationships among latent variables more accurately by considering measurement error, and examine several interdependent multiple regressions concurrently. In the current study, safety climate and safety performance factors are latent variables that could not be directly observed. With SEM, a proposed measurement model composed of several safety climate and safety performance factors was examined, and a hypothetical structural model considering their relationships was developed and examined. AMOS version 17.0 was used in the current study. For model estimation, maximum likelihood method was applied. The SEM model was tested in two stages of verifying the measurement model and verifying the structural model. Internal validity and reliability of the model was assessed with calculating average variance extracted (AVE) and construct reliability (CR). A value over 0.50 of AVE and a value over 0.70 of CR suggest good validity and reliability, respectively [36]. Because of the model complexity, internal validity and reliability was firstly accessed within every construct, and then in an aggregated measurement model [37]. For model evaluation, a number of frequently-used fit indices were adopted in the current study, including the ratio of model chi-square to the degrees of freedom (χ^2^/df), root mean square error of approximation (RMSEA), goodness-of-fit (GFI), adjusted goodness-of-fit (AGFI), Tucker–Lewis index (TLI) and comparative fit index (CFI). A χ^2^/df value less than 5 indicates an acceptable model fit to the data. RMESA values of less than 0.05 indicate a good fit, whereas values ranging from 0.05 to 0.08 are acceptable. GFI, AGFI, TLF, and CFI all range from 0 to 1. Values over 0.80 are considered to be acceptable model fit to the data. Mediations were also considered in SEM. The bootstrap method was selected in AMOS version 17.0. The mediation effect exists if the bias-corrected interval for the indirect effect does not include zero. If the bias-corrected interval for the direct effect includes zero, full mediation effect exists; if not, partial mediation effect exists [38].

## 4. Results

### 4.1. The Samples

A total of 1490 questionnaires were distributed; 1120 completed questionnaires were returned for a response rate of 75.2%. After deleting the extreme and missing values, 1030 questionnaires were used for analysis. Overall, approximately 20.4% of the participants were Chinese while the others were Vietnamese (79.6%). Approximately 83.7% of the participants were male and 71.0% were married. The largest group among the participants in terms of age was individuals from 21 to 30 years old (62.2%), while the most common number of family members supported was two (26.0%). Most of the participants had a junior middle school level (31.4%). The range of 1 to 5 years was generally common (64.0%) for the participants’ job tenure. Approximately 39.0% of the participants had a smoking habit, while 42.5% had a drinking habit.

### 4.2. Exploratory Factor Analysis

Around half of the data were used to conduct EFA in the SPSS 17.0 for Windows software package. For EFA on safety climate, the results showed that Kaiser-Mayer-Olkin (KMO) measure of sampling accuracy was 0.891 and Barlett’s test of sphericity was significant (*p* < 0.001), indicating that the data were appropriate for factor analysis [39]. The 38 safety climate items were subjected to a factor analysis with PCA extraction and Oblimin rotation method. This yielded an interpretable result of four factors using the parallel analysis and explained 56.93% of variance. Factors 1, 2, 3, and 4 explained 23.19%, 16.09%, 10.84% and 6.81% of the variance, respectively.

As shown in Table 1, the final result includes 27 items with factor loadings above 0.50 on one of these factors. The factor loadings of each item and the percentage of variance explained by each factor are also shown in this table. The first factor was interpreted as top management commitment (TMC) and it consisted of 10 items from OSC indicating the attitude to safety of top management level in the organization. The explanation of the second factor was coworkers’ caring and communication (CCC) and it included eight items from CSC which reflect coworkers’ opinion on communicating with other workers and their willingness to help other workers. The third factor was explained as coworkers’ role models (CRM) since it comprised four items from CSC indicating coworkers’ safety behaviors that could provide a fine example to other workers. The interpretation of the fourth factor was supervisors’ expectation (SE) and it contained five items from SSC focusing on supervisor’s attitude to construction safety. All the factors included more than four items, and the Cronbach’s alpha coefficients for these four factors were 0.961, 0.887, 0.897, and 0.890, respectively, which were all above 0.70 and considered to be acceptable [40,41]. In summary, TMC and SE were identified to be factors that represent OSC and SSC, respectively, while CCC and CRM were identified as factors to denote CSC. That is to say, OSC and SSC were explained by one factor each, while CSC was explained by two factors.

For EFA on safety performance, the results showed Kaiser-Mayer-Olkin (KMO) measure of sampling accuracy was 0.860 and Barlett’s test of sphericity was significant (*p* < 0.001). The safety performance (SP) items were also subjected to a factor analysis with PCA extraction and Oblimin rotation method. This yielded an interpretable result of one factor and explained 56.04% of variance. As shown in Table 2, the final result includes six items with factor loadings above 0.50 on this factor.

**Figure 2:**
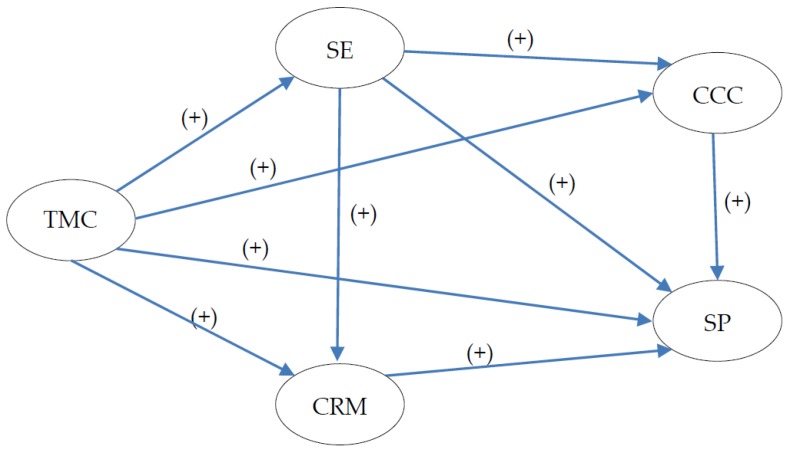
Further development of research model and hypotheses. TMC: top management commitment, SE: supervisors’ expectations, CRM: coworkers’ role models, CCC: coworkers’ caring and communication, SP: safety performance.

As CCC and CRM are two separate safety climate factors on the CSC level, the proposed research model in Figure 1 could be further developed as Figure 2.

### 4.3. Measurement Model Assessment

A proposed measurement model composed of TMC, SE, CCC, CRM, and SP was examined in the current study. The remaining half of the data was submitted to SEM in the Analysis of Moment Structures (AMOS) version 17.0. Table 3 shows the empirically tested results of the multilevel safety climate and safety performance measurement model with standardized parameter estimates. The analysis retained items with factor loadings larger than 0.50 [36]. OSC1, OSC6, OSC7, and OSC15 were thus removed from the factor of TMC. CSC7 and CSC3 were removed from the factor of CCC, and CSC1 was removed from the factor of CRM. SPart. 2 and SPart. 3 were removed from the factor of SP. The results showed that all values of CR for the four constructs were more than 0.70, thereby advising a satisfactory level of construct reliability. The values of AVE were all around or higher than 0.50, suggesting a satisfactory level of construct validity [36]. According to Table 4, the selected model fit indices were all at the acceptable level for the measurement model (χ^2^/df = 4.091, GFI = 0.879, AGFI = 0.849, RMSEA = 0.073, TLI = 0.898, and CFI = 0.911). All paths from the observed variables to the latent factors were significant.

### 4.4. Structural Model Assessment

**Figure 3:**
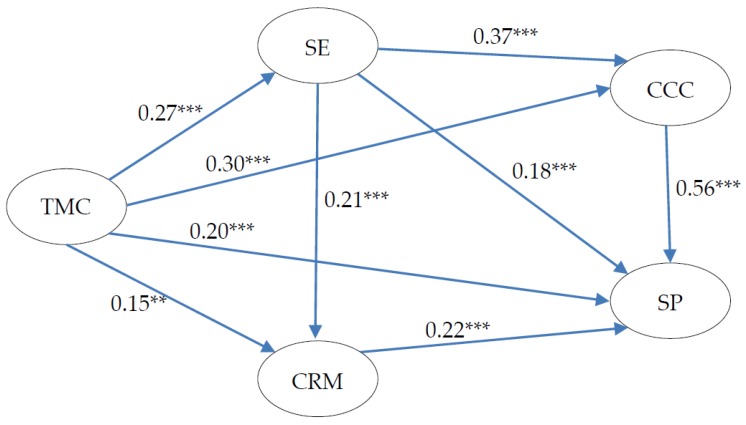
Testing results of the research model. ** *p* < 0.01, *** *p* < 0.001.

According to Table 4, the model fit indices of the whole structural model were at the acceptable level (χ^2^/df = 4.595, GFI = 0.864, AGFI = 0.833, RMSEA = 0.079, TLI = 0.882, and CFI = 0.896). The testing results of the structural model were shown in Figure 3. Numbers on the arrows represent the path coefficients, which indicate the strength of the relationships among latent variables.

H1: The OSC factor was confirmed to be positively related to SSC factor and CSC factors. H1 was supported. TMC had a strong statistically significant positive relationship with SE (β = 0.27, *p* < 0.001). In addition, TMC was confirmed to have a significantly positive relationship with the two CSC factors: CCC (β = 0.30, *p* < 0.001) and CRM (β = 0.15, *p* < 0.01).

H2: The SSC factor was confirmed to mediate the relationship between OSC factor and CSC factors. H2 was supported. Table 5 shows the effect paths from the OSC factor on CSC factors, OSC factor on SP, and SSC factor on SP. The indirect effect of TMC on CCC was positive and significant (indirect effect = 0.083, *p* < 0.001), and the indirect effect of TMC on CRM was also positive and significant (indirect effect = 0.042, *p* < 0.001). Considering the existence of significant direct effect of TMC on CCC (direct effect = 0.258, *p* < 0.001) and TMC on CRM (direct effect = 0.113, *p* < 0.05), partially mediated relationships were considered between TMC and CCC, and between TMC and CRM. Partial mediation means that the mediator explains part of the relationship between the two constructs, implying a significant relationship between the mediator and the dependent variable, as well as some direct relationship between the independent and dependent variable.

H3: The SSC factor was confirmed to mediate the relationship between the OSC factor and SP. H3 was supported. The indirect effect of TMC on SP was positive and significant (indirect effect = 0.091, *p* < 0.001). Considering the existence of significant direct effect of TMC on SP (direct effect = 0.325, *p* < 0.001), a partially mediated relationship was considered between TMC and SP.

H4: The CSC factors were confirmed to mediate the relationship between the OSC factor and SP. H4 was supported. The indirect effects of TMC on SP via CCC (indirect effect = 0.243, *p* < 0.001) and CRM (indirect effect = 0.081, *p* < 0.001) were positive and significant. Considering the existence of the significant direct effect of TMC on SP via CCC (direct effect = 0.184, *p* < 0.001) and CRM (direct effect = 0.328, *p* < 0.001), partially mediated relationships were considered between the OSC factor and SP by CSC factors.

H5: The CSC factors were confirmed to mediate the relationship between the SSC factor and SP. H5 was supported. The indirect effects of SE on SP via CCC (indirect effect = 0.268, *p* < 0.001) and CRM (indirect effect = 0.096, *p* < 0.001) were positive and significant. Considering the existence of the significant direct effect of SE on SP via CCC (direct effect = 0.170, *p* < 0.001) and CRM (direct effect = 0.323, *p* < 0.001), partially mediated relationships were considered between the SSC factor and SP by CSC factors.

## 5. Discussion

As hypothesized, the OSC factor (TMC) is positively and significantly related to the SSC factor (SE) and CSC factors (CCC and CRM). The SSC factor (SE) could partially mediate the relationship between the OSC factor (TMC) and CSC factors (CCC and CRM), as well as the relationship between the OSC factor (TMC) and SP. CSC factors (CCC and CRM) play a statistically significant partial mediation role in the relationship between the OSC factor (TMC) and SP, and the relationship between the SSC factor (SE) and SP.

Top management commitment to safety is crucial for enhancing safety management in construction projects [16,42,43]. Management commitment plays an important role in creating a positive safety culture by considering safety as an integrated component of the production system from the top, rather than thinking of it as an independent part of the production process [44]. Top management commitment could be explained as the management of allocating resources and time, site inspections and risk assessments, and participation in safety meetings [42]. Participation of top management in safety committees and the empowerment of safety officers are deemed to be critically important [44]. The management should ‘walk the talk’ and make efforts to actively and consistently support safety. The workers tend to perceive the real attitude from the management layer, and follow the example and actions of them accordingly. The management is thus incumbent on establishing a positive and practical safety standard for the workers [45]. In construction projects, top management should make special efforts to overcome the particularly hazardous environment and make up for the physical and psychological distance between the headquarters and projects [12].

The identified SSC factor (SE) was confirmed to mediate the relationship between the OSC factor (TMC) and safety performance. This result is similar to the results of Zohar and Luria [19]. Safety policies, procedures, and regulations, which are formulated at the organization level, provide strategic and tactical rules for safety management. Safety practices, which relate to the implementation of these policies, procedures, and regulations, are put into action at the group level. Safety climate could both be formed from top management’s policy actions and front-line supervisors’ practical actions. Communicating with supervisors shows workers the true priority of safety through handling conflicting demands between productivity and safety [11]. In construction projects, the site is full of continuous changes and immediate actions, and subcontracting is extremely common. The projects might be distant from headquarters and the front-line workers may often hardly see the top management. The temporary nature of construction projects, the characteristics of construction procedures, and the physical distance from headquarters could further reduce the direct impact of organizational factors on safety performance [2]. On the commitment of top management commitment to safety climate could be formed through site supervisors’ practical attitudes and actions. As such, effective communication between site supervisors and front-line workers should be set up to make workers understand company safety regulations more easily and to improve safety performance accordingly.

CSC factors (CCC and CRM) were confirmed to partially mediate the relationship between the OSC factor (TMC) and safety performance, and the relationship between the SSC factor (SE) and safety performance. The mediation effect of CCC was relatively stronger than SE for the relationship between TMC and SP, and this result echoed the study of Tucker et al. [46] and Chiaburu and Harrison [47], which suggested that coworker support could predict many employee performances better than leader support could. Coworkers caring and communication and the effect of role models have great effects on ensuring workers’ safety performance. Coworkers’ attitudes and actions to safety are supposed to influence safety performance because they provide beliefs about what kind of actions are socially acceptable within a workgroup or organization. CSC factors mediated the relationship between SE and SP, as well as TMC and SP for the following reasons. For SE, which is an SSC factor, coworkers support supervisors to reduce pressures to communicate and access to resources, as well as enhance supervisors’ awareness of self-efficacy to engage in safety leadership [48]. For TMC, which is an OSC factor, attitudes and actions regarding safety may also originate from their own perceptions of management’s commitment to safety [31]. It is thus important to create a friendly relationship among coworkers. Beyond taking personal responsibility, workers should also be educated to promote a sense of responsibility for coworkers’ safety in order to create a safer work environment. Workers should be supported to remind their coworkers of safety by caring, communicating, and playing the part of role models.

## 6. Conclusions

The current study explores the underlying mechanisms of the relationship between safety climate and safety performance. To be specific, it investigates how particular safety climate factors of one level affect those of other levels, and then affect safety performance from the top down. According to the EFA results, TMC and SE were identified as factors to represent OSC and SSC, respectively, and CCC and CRM were identified as factors to denote CSC. After verifying these identified factors in an integrated model in SEM, all five proposed hypotheses were tested and supported. The OSC factor (TMC) is positively and significantly related to the SSC factor (SE) and CSC factors (CCC and CRM). The SSC factor (SE) could partially mediate the relationship between the OSC factor (TMC) and CSC factors (CCC and CRM), as well as the relationship between the OSC factor (TMC) and SP. CSC factors (CCC and CRM) play a statistically significant partial mediation role in the relationship between the OSC factor (TMC) and SP, and the relationship between the SSC factor (SE) and SP.

The findings of this study provide practical implications. A positive safety culture should be established both at the organizational level and the group level. Efforts from top management, supervisors, and coworkers should be provided to safety management. First, the top management should ‘walk the talk’ and make efforts to actively and consistently support safety. Second, effective communication between site supervisors and front-line workers should be set up to make workers understand company safety regulations more easily and to improve safety performance accordingly. Third, workers should be educated to promote a sense of responsibility for coworkers’ safety to create a safer work environment and be supported to remind their coworkers of safety by caring, communicating, and playing their part as role models.

This study has several limitations. First, similar to any other self-reported survey, common method biases may exist in the current research. To control and alleviate their effects, several techniques have been adopted. At the questionnaire design stage, a number of reverse-coded items were included in the scales to reduce the possible effects of response pattern bias. At the questionnaire administration stage, the participants were notified that their responses were anonymous and confidential, and answers were not right or wrong, and therefore they should answer questions as frankly as possible. Second, this study used self-reported questionnaires to measure safety performance. It could obtain objective measures of safety performance to find out how the integrated multilevel safety climate works on them in the future. Third, the current study does not consider some of the important personal particulars of workers (e.g., trade information). Future research should consider additional information of workers (e.g., trade types) to further improve the research findings. Future work should also validate the research findings in the current study by getting data from a larger number of organizations.

## Figures and Tables

**Table 1:** Exploratory factor analysis of safety climate.

Construct	Code	Factor Loading	Cronbach’s Alpha	Variance Explained (%)
Top Management Commitment (TMC)	OSC2	0.886	0.961	23.19
	OSC1	0.873		
	OSC6	0.872		
	OSC15	0.866		
	OSC12	0.853		
	OSC7	0.852		
	OSC16	0.850		
	OSC3	0.846		
	OSC8	0.809		
	OSC5	0.807		
Coworkers’ Caring and Communication (CCC)	CSC10	0.795	0.887	16.09
	CSC11	0.780		
	CSC6	0.749		
	CSC7	0.745		
	CSC9	0.745		
	CSC4	0.728		
	CSC5	0.726		
	CSC3	0.662		
Coworkers’ Role Models (CRM)	CSC8	0.834	0.897	10.84
	CSC2	0.830		
	CSC12	0.814		
	CSC1	0.699		
Supervisors’ Expectation (SE)	SSC9	0.874	0.890	6.81
	SSC10	0.869		
	SSC6	0.799		
	SSC8	0.786		
	SSC7	0.701		

OSC: organizational safety climate, CSC: coworker safety climate, SSC: supervisor safety climate.

**Table 2:** Exploratory factor analysis of safety performance.

Construct	Code	Factor Loading	Variance Explained (%)
Safety Performance (SP)	SPart.1	0.777	56.04
	SComp.3	0.777	
	SComp.2	0.759	
	SComp.1	0.758	
	SPart.3	0.743	
	SPart.2	0.673	

**Table 3:** Measurement model evaluation.

Construct	Code	Loading	AVE (Average Variance Extracted)	Composite Reliability
TMC (OSC)	OSC2	0.743	0.704	0.934
	OSC3	0.873		
	OSC5	0.863		
	OSC8	0.868		
	OSC12	0.845		
	OSC16	0.836		
SE (SSC)	SSC6	0.651	0.635	0.895
	SSC7	0.699		
	SSC8	0.805		
	SSC9	0.877		
	SSC10	0.919		
CCC (CSC-Factor 1)	CSC4	0.696	0.504	0.859
	CSC5	0.709		
	CSC6	0.708		
	CSC9	0.679		
	CSC10	0.736		
	CSC11	0.729		
CRM (CSC-Factor 2)	CSC2	0.793	0.525	0.766
	CSC8	0.602		
	CSC12	0.765		
SP	SComp. 1	0.677	0.492	0.794
	SComp. 2	0.727		
	SComp. 3	0.766		
	SPart. 1	0.627		

**Table 4:** Goodness-of-fit indexes for measurement and structural models.

Model	χ^2^	χ^2^/DF	GFI	AGFI	RMSEA	TLI	CFI
Measurement	989.918	4.091	0.879	0.849	0.073	0.898	0.911
Structural	1116.697	4.595	0.864	0.833	0.079	0.882	0.896

DF: degrees of freedom, GFI: goodness-of-fit, AGFI: adjusted goodness-of-fit, RMSEA: root mean square error of approximation, TLI: Tucker–Lewis index, CFI: comparative fit index.

**Table 5:** Breakdown of effect paths.

Effect Paths	Total Effect	Indirect Effect	Direct Effect	Type
Effect of OSC on CSC				
TMC→SE→CCC	0.341 ***, (0.249, 0.440)	0.083 ***, (0.047, 0.128)	0.258 ***, (0.176, 0.354)	Partial Mediation
TMC→SE→CRM	0.155 ***, (0.079, 0.229)	0.042 ***, (0.014, 0.079)	0.113 *, (0.031, 0.192)	Partial Mediation
Effect of OSC on SP				
TMC→SE→SP	0.416 ***, (0.328, 0.522)	0.091 ***, (0.055, 0.136)	0.325 ***, (0.244, 0.417)	Partial Mediation
TMC→CCC→SP	0.427 ***, (0.339, 0.529)	0.243 ***, (0.173, 0.337)	0.184 ***, (0.118, 0.257)	Partial Mediation
TMC→CRM→SP	0.409 ***, (0.319, 0.512)	0.081 ***, (0.042, 0.128)	0.328 ***, (0.249, 0.431)	Partial Mediation
Effect of SSC on SP				
SE→CCC→SP	0.438 ***, (0.336, 0.561)	0.268 ***, (0.188, 0.364)	0.170 ***, (0.089, 0.259)	Partial Mediation
SE→CRM→SP	0.419 ***, (0.317, 0.532)	0.096 ***, (0.050, 0.155)	0.323 ***, (0.234, 0.421)	Partial Mediation

* *p* < 0.05, *** *p* < 0.001.

## References

[1] Mohamed S. (2002). Safety Climate in Construction Site Environments. J. Constr. Eng. Manag..

[2] Swuste P., Frijters A., Guldenmund F. (2012). Is it possible to influence safety in the building sector? A literature review extending from 1980 until the present. Saf. Sci..

[3] Sunindijo R.Y., Zou P.X.W. (2012). Political Skill for Developing Construction Safety Climate. J. Constr. Eng. Manag..

[4] Huang X., Hinze J. (2006). Owner’s role in construction safety. J. Constr. Eng. Manag..

[5] Neal A., Griffin M.A., Hart P.M. (2000). The impact of organizational climate on safety climate and individual behavior. Saf. Sci..

[6] Clarke S. (2006). The relationship between safety climate and safety performance: A meta-analytic review. J. Occup. Health Psychol..

[7] Hon C.K.H., Chan A.P.C., Yam M.C.H. (2014). Relationships between safety climate and safety performance of building repair, maintenance, minor alteration, and addition (RMAA) works. Saf. Sci..

[8] Barbaranelli C., Petitta L., Probst T.M. (2015). Does safety climate predict safety performance in Italy and the USA? Cross-cultural validation of a theoretical model of safety climate. Accid. Anal. Prev..

[9] Desai V.M., Roberts K.H., Ciavarelli A.P. (2006). The relationship between safety climate and recent accidents: Behavioral learning and cognitive attributions. Hum. Factors.

[10] Pinto A. (2014). QRAM a Qualitative Occupational Safety Risk Assessment Model for the construction industry that incorporate uncertainties by the use of fuzzy sets. Saf. Sci..

[11] Sparer E.H., Murphy L.A., Taylor K.M., Dennerlein J.T. (2013). Correlation between safety climate and contractor safety assessment programs in construction. Am. J. Ind. Med..

[12] Meliá J.L., Mearns K., Silva S.A., Lima M.L. (2008). Safety climate responses and the perceived risk of accidents in the construction industry. Saf. Sci..

[13] Brondino M., Silva S.A., Pasini M. (2012). Multilevel approach to organizational and group safety climate and safety performance: Co-workers as the missing link. Saf. Sci..

[14] Fang D., Chen Y., Wong L. (2006). Safety climate in construction industry: A case study in Hong Kong. J. Constr. Eng. Manag..

[15] Dedobbeleer N., Béland F. (1991). A safety climate measure for construction sites. J. Saf. Res..

[16] Hon C.K.H., Chan A.P.C., Yam M.C.H. (2013). Determining Safety Climate Factors in the Repair, Maintenance, Minor Alteration, and Addition Sector of Hong Kong. J. Constr. Eng. Manag..

[17] Lingard H.C., Cooke T., Blismas N. (2010). Properties of group safety climate in construction: The development and evaluation of a typology. Constr. Manag. Econ..

[18] Zohar D. (2000). A Group-Level Model of Safety Climate: Testing the Effect of Group Climate on Microaccidents in Manufacturing Jobs. J. Appl. Psychol..

[19] Zohar D., Luria G. (2005). A multilevel model of safety climate: Cross-level relationships between organization and group-level climates. J. Appl. Psychol..

[20] Lingard H.C., Cooke T., Blismas N. (2009). Group-level safety climate in the Australian construction industry: Within-group homogeneity and between-group differences in road construction and maintenance. Constr. Manag. Econ..

[21] Lingard H., Cooke T., Blismas N. (2011). Coworkers’ response to occupational health and safety: An overlooked dimension of group-level safety climate in the construction industry?. Eng. Constr. Archit. Manag..

[22] Christian M.S., Bradley J.C., Wallace J.C., Burke M.J. (2009). Workplace safety: A meta-analysis of the roles of person and situation factors. J. Appl. Psychol..

[23] Grabowski M., Ayyalasomayajula P., Merrick J. (2007). Denise Mccafferty Accident precursors and safety nets: Leading indicators of tanker operations safety. Marit. Policy Manag..

[24] Johnson S.E. (2007). The predictive validity of safety climate. J. Saf. Res..

[25] Neal A., Griffin M.A. (2006). A study of the lagged relationships among safety climate, safety motivation, safety behavior, and accidents at the individual and group levels. J. Appl. Psychol..

[26] Griffin M.A., Neal A. (2000). Perceptions of safety at work: A framework for linking safety climate to safety performance, knowledge, and motivation. J. Occup. Health Psychol..

[27] Neal A., Griffin M.A. (2004). Safety Climate and Safety at Work.

[28] Siu O.-L., Phillips D.R., Leung T.-W. (2004). Safety climate and safety performance among construction workers in Hong Kong. Accid. Anal. Prev..

[29] Huang Y.H., Ho M., Smith G.S., Chen P.Y. (2006). Safety climate and self-reported injury: Assessing the mediating role of employee safety control. Accid. Anal. Prev..

[30] Guo B.H.W., Yiu T.W., González V.A. (2016). Predicting safety behavior in the construction industry: Development and test of an integrative model. Saf. Sci..

[31] Morrow S.L., McGonagle A.K., Dove-Steinkamp M.L., Walker C.T., Marmet M., Barnes-Farrell J.L. (2010). Relationships between psychological safety climate facets and safety behavior in the rail industry: A dominance analysis. Accid. Anal. Prev..

[32] Thompson B. (2004). Exploratory and Confirmatory factor Analysis: Understanding Concepts and Applications.

[33] Choudhry R., Fang D., Lingard H. (2009). Measuring Safety Climate of a Construction Company. J. Constr. Eng. Manag..

[34] Pallant J. (2007). A Step by Step Guide to Data Analysis Using SPSS for Windows Version 15.

[35] Lingard H., Sublet A. (2002). The impact of job and organizational demands on marital or relationship satisfaction and conflict among Australian civil engineers. Constr. Manag. Econ..

[36] Hair J.F., Black W.C., Babin B.J., Anderson R.E. (2010). Multivariate Data Analysis.

[37] Shen Y. (2013). An Investigation of Safety Climate on Hong Kong Construction Sites. Ph.D. Thesis.

[38] Shrout P.E., Bolger N. (2002). Mediation in experimental and nonexperimental studies: New procedures and recommendations. Psychol. Methods.

[39] Kaiser H.F. (1974). An index of factorial simplicity. Psychometrika.

[40] Litwin M.S. (1995). How to Measure Survey Reliability and Validity.

[41] Zhou Q., Fang D., Mohamed S. (2010). Safety climate improvement: Case study in a Chinese construction company. J. Constr. Eng. Manag..

[42] Choudhry R.M., Fang D., Mohamed S. (2007). The nature of safety culture: A survey of the state-of-the-art. Saf. Sci..

[43] Lingard H., Cooke T., Blismas N. (2012). Do Perceptions of Supervisors’ Safety Responses Mediate the Relationship between Perceptions of the Organizational Safety Climate and Incident Rates in the Construction Supply Chain?. J. Constr. Eng. Manag..

[44] Zohar D. (1980). Safety climate in industrial organizations: Theoretical and applied implications. J. Appl. Psychol..

[45] Aksorn T., Hadikusumo B.H.W. (2008). Critical success factors influencing safety program performance in Thai construction projects. Saf. Sci..

[46] Tucker S., Chmiel N., Turner N., Hershcovis M.S., Stride C.B. (2008). Perceived organizational support for safety and employee safety voice: The mediating role of coworker support for safety. J. Occup. Health Psychol..

[47] Chiaburu D.S., Harrison D.A. (2008). Do peers make the place? Conceptual synthesis and meta-analysis of coworker effects on perceptions, attitudes, OCBs, and performance. J. Appl. Psychol..

[48] Conchie S.M., Moon S., Duncan M. (2013). Supervisors’ engagement in safety leadership: Factors that help and hinder. Saf. Sci..

